# Unilateral testicular metastasis of prostate cancer

**DOI:** 10.4322/acr.2024.507

**Published:** 2024-07-12

**Authors:** Ravi Hari Phulware, Gayathri K S, Gaurav Rajendra Shirsath, Amit Gupta

**Affiliations:** 1 All India Institute of Medical Sciences (AIIMS), Department of Pathology & Laboratory Medicine, Rishikesh, Uttarakhand, India; 2 All India Institute of Medical Sciences (AIIMS), Department of General Surgery, Rishikesh, Uttarakhand, India

**Keywords:** Neoplasm Metastasis, Prostate-Specific Antigen, Prostatic Neoplasms, Testicular Neoplasms, Testis

Prostatic adenocarcinoma is the most common type of cancer in men, with subsequent lesions most commonly occurring in the lymph nodes, bones, and lungs.^1,2^ We discuss the clinical case of a 59-year-old man who presented after radical prostatectomy for prostate cancer with a unilateral metastasis in the right testis. Metastases to the testis are uncommon, occurring in less than 4% of cases.^2,3^ This kind of metastasis is usually unilateral, manifesting as a palpable testicular mass, and rarely involves both the testis and the epididymis.^1,2^

Secondary neoplasms of the testis are found in around 2.5% of autopsies, including nonneoplastic fatalities.^2^ Most secondary testicular metastases develop from distant primary locations, the most prevalent of which are the lung, prostate, and gastrointestinal tract.^2^

Testicular metastases can occur in up to 4% of all prostate cancer cases and are frequently discovered by chance following orchiectomy therapy for advanced disease.^4^ In general, advanced prostate cancer metastases to the pelvic lymph nodes, bones, lungs, and liver are common; however, few of these patients have clinically evident testicular metastasis.^4,5^

Secondary neoplasms of the testis are uncommon, with a reported rate of 0.02-2.5%, except leukemia and lymphoma infiltration.^2,3^ The prostate is the most common site of testicular metastases (15%), followed by the lung, melanomas, skin, colon, and kidney.^1,2^ However, most testicular metastases of prostate cancer were discovered after examining a significant number of testes from patients who had tumors removed during therapeutic orchiectomies.^2,4^

Lung cancer (43%), malignant melanoma (20%), pancreatic cancer (10%), and prostate cancer (10%) are among cancers that can spread to the testes.^4,5^ However, the great majority of metastases are lesions detected by chance following an autopsy or bilateral orchiectomy for Prostatic Cancer hormonal treatment.^3^ A testicular tumor revealing clinical recurrence is highly unusual.

Bubendorf et al.^1^ discovered testicular metastases in just 0.5% of 1,589 prostate carcinoma autopsy reports. He also suggests a backward metastatic channel through prostate veins in addition to the typical hematogenous tumor spread via the vena cava. Overall, four routes have been hypothesized for the propagation of the lesions to the testis: retrograde venous extension, retrograde lymphatic extension, arterial embolism, and through the lumen of the vas deferens.^1^

Patients with symptomatic isolated post-prostatectomy testicular metastases can live for a surprisingly long time after orchidectomy without further development. This phenomenon may be linked to cytoreduction.^1,2^ The ability to manage the malignant process locally is an undeniable clinical benefit for patients undergoing orchiectomy. Given that this method of treatment is easy, safe, and associated with few problems, all patients with isolated prostatic cancer testicular metastases should be considered candidates for metastasectomy.^4,5^

The histological features of prostate cancer testicular metastases are similar to those of original prostate tumors; however, histology may show a more aggressive phenotype with a significant probability of future disease spreading and, thus, worse survival.^3,4^ Weitzner^4^ found that patients with newly diagnosed testicular metastases from prostate cancer had a median survival of roughly 12 months.^1,2^ Other studies, on the other hand, have documented survival of more than two years without biochemical relapses. As a result, the predictive impact of testicular metastases from prostate cancer is yet unknown, owing to the rarity of the incidence.^2^

Testicular metastasis from prostate carcinoma is an uncommon manifestation of advanced disease.^2^ It often presents with testicular pain, swelling, or a palpable mass and can mimic primary testicular neoplasms. Clinicians should maintain a high index of suspicion, especially in patients with a history of prostate carcinoma. Prompt diagnosis through imaging studies and histopathological confirmation is essential for appropriate management and prognosis.^3,4^

This case underscores the importance of considering testicular metastasis in the differential diagnosis of testicular masses, particularly in patients with a history of prostate carcinoma. A multidisciplinary approach is crucial for optimal management and improving patient outcomes. Early recognition and timely intervention are paramount in managing metastatic disease and improving quality of life.

**Figure 1:**
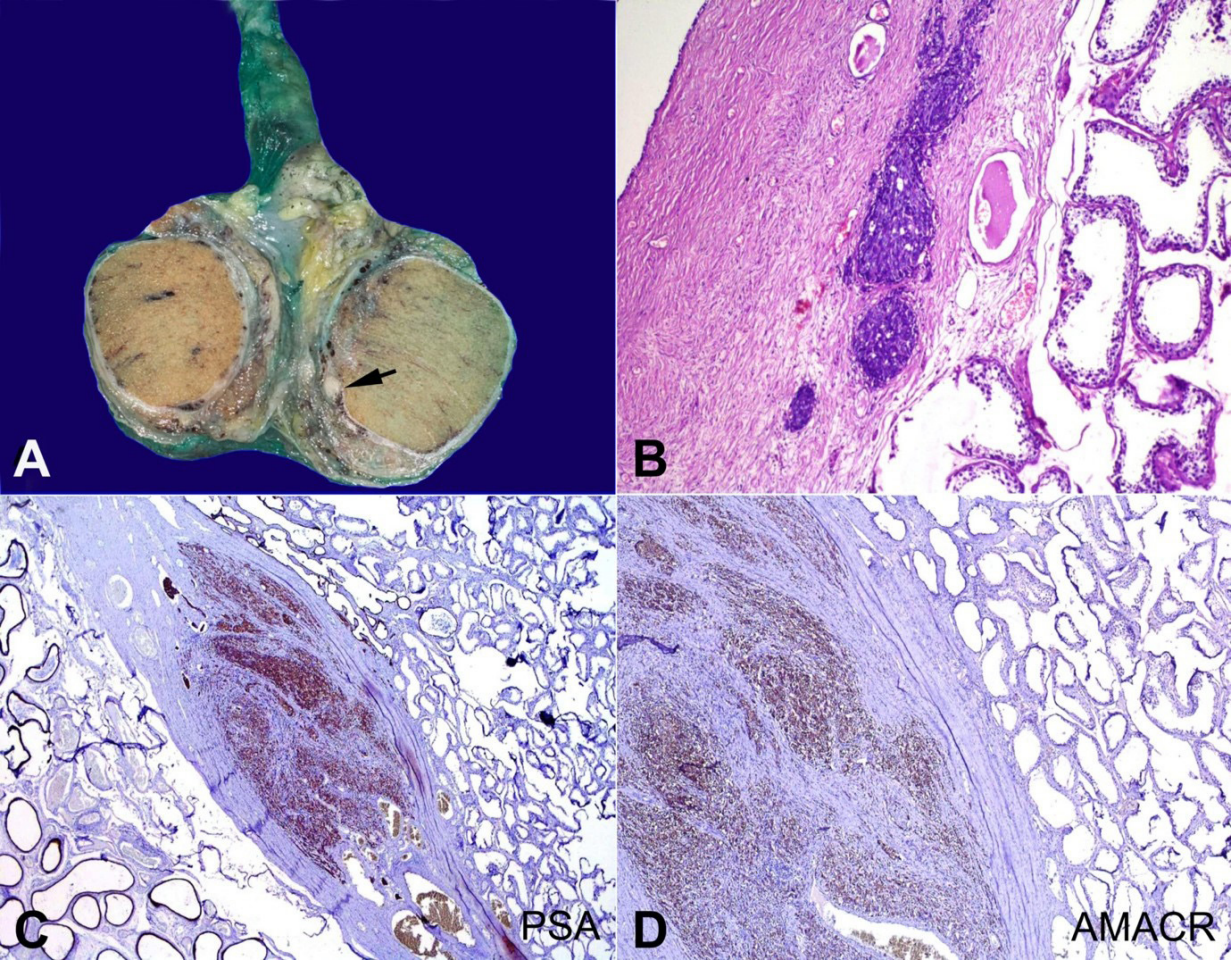
**A –** Gross view of the right orchidectomy showing a small nodular metastatic deposit in tunica albuginea from the prostate cancer; **B**, **C** and **D** are photomicrographs of the tumor and testicle parenchyma; **B –** shows normal testicular seminiferous tubules along with presence of metastatic tumor deposit in the wall of testis (Tunica albuginea) (H&E x100); **C –** These tumor cells are immunopositive for prostate specific antigen (PSA 40X); **D –** The tumor cells are also positive for Alpha-methylacyl-CoA racemase (AMACR 200X).

Figure 1 refers to a 59-year-old male patient presented to our hospital with an elevated serum prostate-specific antigen (PSA) level of >100 ng/ml. He had been on regular follow-up with urology for surveillance of prostate cancer recurrence. He had undergone a radical prostatectomy two years ago with Gleason grade group 5. Scrotal ultrasound showed a hypoechoic mass involving the right testicle with increased vascularity, suggesting a neoplastic lesion. Hence, the patient was planned for a bilateral orchidectomy. The patient underwent bilateral inguinal orchiectomy without any complications. Macroscopic examination of the right resected testis revealed a solid yellowish-white tumor of 0.5 × 0.5 cm. Histopathological analysis showed that the right testis was infiltrated by metastatic adenocarcinoma. Immunohistochemical examination revealed that tumor cells were diffusely positive for PSA and Alpha (α)-methylacyl-CoA racemase (AMACR). The patient was diagnosed with right testicular metastasis of Prostatic Carcinoma. The patient had an uneventful postoperative recovery. The patient was scheduled for regular follow-up appointments to monitor disease progression. Serial PSA levels and imaging studies were planned to look for disease status.
